# The role of vulpinic acid as a natural compound in the regulation of breast cancer-associated miRNAs

**DOI:** 10.1186/s40659-021-00360-4

**Published:** 2021-11-07

**Authors:** Demet Cansaran-Duman, Sevcan Yangın, Betül Çolak

**Affiliations:** Ankara University, Biotechnology Institute, Keçiören, Ankara, 06135 Turkey

**Keywords:** Vulpinic acid, microRNA analysis, Breast cancer

## Abstract

**Background:**

Breast cancer is the most frequently diagnosed cancer, and no effective treatment solution has yet been found. The number of studies based on the research of novel natural compounds in the treatment of breast cancer has been increasing in recent years. The anticancer properties of natural compounds are related to the regulation of microRNA (miRNA) expression. Therefore, changing the profile of miRNAs with the use of natural products is very important in cancer treatment. However, the role of vulpinic acid and related miRNAs in breast cancer progression remains unknown. Vulpinic acid, methyl (as2*E*)-2-(3-hydroxy-5-oxo-4-phenylfuran-2-ylidene)-2 phenylacetate, is a natural product extracted from the lichen species and shows an anticancer effect on different cancer cells.

**Methods:**

This study examines the effects of vulpinic acid on the miRNA levels of breast cancer (MCF-7) cells and its relationship with cell proliferation and apoptosis levels. The antiproliferative effect of vulpinic acid was screened against MCF-7 breast cancer cells and MCF-12A breast epithelial cells using the xCELLigence real-time cell analysis system. We analyzed the altered miRNA expression profile in MCF-7 breast cancer cells versus MCF-12A cells following their response to vulpinic acid through microarray analysis. The microarray analysis results were confirmed through quantitative real-time PCR and bioinformatics analysis.

**Results:**

The results of the miRNA array and bioinformatic analyses demonstrated that 12 miRNAs were specifically responsive to vulpinic acid in MCF-7 breast cancer cells. This is the first study to reveal that vulpinic acid inhibits the expression of 12 miRNAs and suppresses breast cancer cell proliferation. The study also revealed that vulpinic acid may downregulate the expression of 12 miRNAs by repressing the *FOXO-3* gene. The miRNA targets were mainly found to play a role in the apoptosis, cell cycle and MAPK pathways. Moreover, Bcl-2, Bax, procaspase-3 and procaspase-9 protein levels were assessed by western blot analysis for validation of apoptosis at the protein level.

**Conclusion:**

This study revealed the molecular mechanisms of vulpinic acid on breast cancer and showed that vulpinic acid regulates apoptosis signaling pathways by decreasing the expression of miRNAs. The miRNA expression patterns illuminate the underlying effect of vulpinic acid in breast cancer treatment.

**Graphical Abstract:**

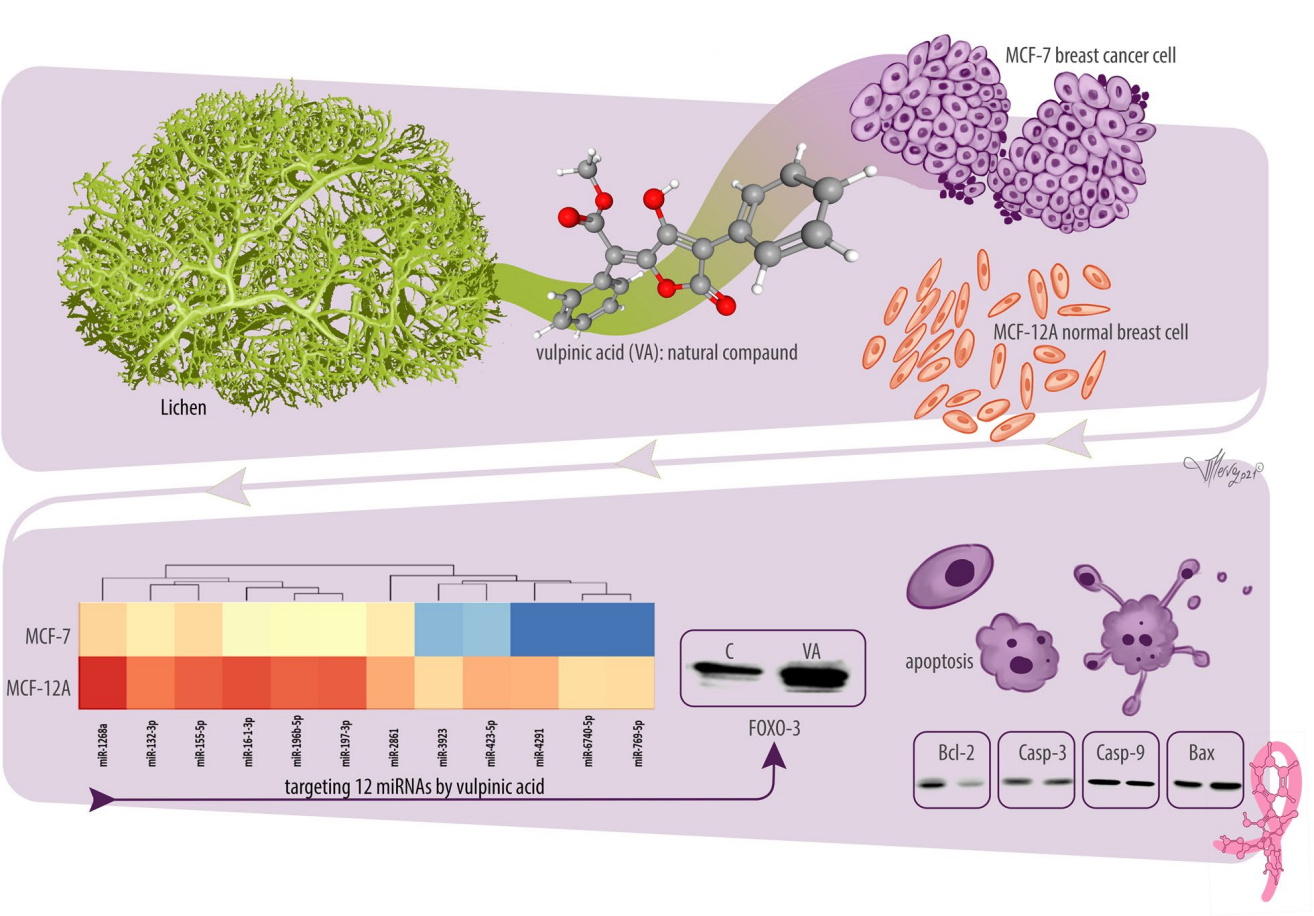

**Supplementary Information:**

The online version contains supplementary material available at 10.1186/s40659-021-00360-4.

## Background

Breast cancer is the most commonly diagnosed and malignant cancer subtype among women [1]. It is a heterogeneous disease with different phenotypes based on gene expression analysis [2]. The identified phenotypes of breast cancer are classified into subtypes: luminal ER positive, HER2 enriched and basal-like [2]. Differently planned treatment options should be carried out for the different subtypes of breast cancer as tumor morphology, size, grade classification and the expression levels of ER, PR and HER2 [3]. Cancer treatment types can be classified into radiation therapy, surgery, endocrine therapy, targeted therapy and chemotherapy [4]. These treatment types can reduce the effect of malignancy but raise the risk of further diseases. Chemotherapy is the most common treatment used for breast cancer. While chemotherapeutics destroy cancer cells, they also damage normal cells. In addition to this side effect, when the patient develops resistance to the highly cytotoxic drug, two or more cytotoxic drugs are mixed, resulting in more physical and psychological side effects for the human [4]. Since chemotherapeutics present significant toxicity for the human body, newly developed breast cancer treatment strategies should be considered. There is thus a particular need to understand the biological mechanisms underlying breast cancer and to develop novel, effective options for the diagnosis, prognosis and treatment of breast cancer.

Natural compounds may have anticancer effects that can be used as chemotherapeutics in breast cancer [5]. In recent years, molecules derived from the natural source of lichen have been shown to have an anticancer effect on cancer cells [6]. Solárová et al. showed the anticancer effect of lichen secondary metabolites with in vivo and in vitro studies [7]. Paluszczak et al. demonstrated that lichens play a significant role in treating cancer subtypes through their anticancer activities [8]. Their study used lichen-derived caperatic acid and physodic acid to treat colon cancer [8]. Vulpinic acid (VA), methyl (2*E*)-2-(3-hydroxy-5-oxo-4-phenylfuran-2-ylidene)-2-phenylacetate, is one of the most promising lichen secondary metabolites that is safe, economical and easily accessible in nature [9]. Vulpinic acid can be considered as a potentially novel candidate molecule for cancer therapy since it has shown antiproliferative, antiangiogenic and anticancer effects [7, 10]. The pathways mainly affected by the application of vulpinic acid to cancer cells are cell cycle, apoptosis, transcription, translation and glycogen metabolism [11]. The cytotoxic and antiproliferative effects of vulpinic acid have been investigated for several cancer cell lines [12, 13]. Kılıç et al. demonstrated the inhibited cell viability of human breast cancer cells (MCF-7, BT-474, MDA-MB-231 and SK-BR-3) through induced apoptosis with vulpinic acid [12]. Remarkably, when the results were evaluated, vulpinic acid had the most antiproliferative effect on MCF-7 breast cancer cells. Another important result obtained was that while vulpinic acid had a very high antiproliferative effect on cancer cells, it did not show a cytotoxic effect on MCF-12A noncancerous breast epithelial cells. However, the previous study also emphasized the antiproliferative effect of vulpinic acid [12]; the anticancer effect of vulpinic acid was not evaluated on the miRNA level.

In recent years, cancer treatment methods have shifted from conventional therapies to treatments associated with small noncoding RNAs. There is the possibility of developing different cancer treatment processes using miRNA-targeted natural compounds. MicroRNAs are small (19–24 nucleotides) noncoding RNAs that play a significant role in gene expression at the translational or posttranslational levels [14]. At the posttranscriptional level, miRNAs regulate gene expression by pairing the sites in 3′ UTR of specific mRNA, which then result in either mRNA degradation or translational inhibition [15]. Through gene expression regulations, miRNAs control critical processes such as apoptosis, proliferation, cell metabolism and angiogenesis of multicellular organisms [16]. Different types of miRNAs are related to specific subtypes of breast cancer pathological characteristics in terms of estrogen and progesterone receptor expression, tumor state, vascular invasion and proliferation information [17]. Circulating miRNAs have their own stability and resistance to endogenous RNAse activity in peripheral blood circulation, which has resulted in them being proposed as diagnostic and prognostic biomarkers [18]. Understanding tumor-specific genetic alterations in miRNA processing machinery contributes solid evidence for relevant pathways in the cellular transformation process [18]. In addition, miRNAs may act as tumor suppressors or oncogenes, which is determined within the cellular context [19]. Studies of the regulation of miRNAs may be the most promising since miRNAs are directly related to cancer phenotypes. In recent years, numerous studies on miRNAs have revealed that miRNAs function as oncogenes or tumor suppressors [20]. Liu and Li et al. showed that trichostatin could upregulate ERά by increasing the miR-204 level in MDA-MB-231 breast cancer cells [21]. Khamisipour et al. determined the therapeutic effect of miR-29a on the expression of genes involved in apoptosis [22]. Several plant compounds have been examined as miRNA expression effectors in different cancer types [23, 24]. There are studies in the literature showing that plant-derived products such as curcumin, artemisinin and epigallocatechin alter miRNA regulation [24–26]. In a previous study, usnic acid was used to determine the role of the natural compound in regulating miRNAs associated with breast cancer [27].

In this study, the anticancer effect of vulpinic acid on MCF-7 (ER + , PR + and HER2-) breast cancer cells and MCF-12A noncancerous breast epithelial cells was determined for the first time using the xCELLigence real-time cell analysis (RTCA) system. We determined the expression profiles of miRNAs that respond to vulpinic acid through miRNA microarray analysis in MCF-7 and MCF-12A cells. Moreover, to determine the specific miRNA profile of vulpinic acid against MCF-7 cancer cells, we extracted the miRNA profile generated in response to the MCF-12A cells from the MCF-7 cancer cell results. The function and pathway analysis of target genes regulated by miRNAs formed as a vulpinic acid response was determined. The results obtained through miRNA array analysis were confirmed by performing bioinformatics analysis, qRT-PCR and western blot analysis. The main objective of this study was to reveal the vulpinic acid mediated regulation of miRNAs and to understand their role in cancer prevention and the development of diagnostics and therapeutics for breast cancer.

## Materials and methods

### Cell culture

MCF-7 human breast cancer cells and MCF-12A human noncancerous breast epithelial cells were obtained from the American Type Culture Collection (ATCC). MCF-7 cells were cultured in Dulbecco’s Modified Eagle Medium (DMEM) (Sigma, USA) supplemented with 10% Fetal Bovine Serum (FBS) (Biological Industries, Israel) and 1% penicillin/streptomycin (Biowest, USA). MCF-12A cells were maintained in DMEM Ham’s F12 Medium (Sigma, USA), which contains 10% FBS, 1% penicillin/streptomycin, 10 μg/ml insulin, 20 ng/ml EGF and 0.5 mg/ml hydrocortisone. The cells were incubated at 37 °C in a humidified atmosphere containing 5% CO_2_.

### Determination of antiproliferative effect using the real-time xCELLigence system

In a previous study, we determined the antiproliferative effect of vulpinic acid on MCF-7 breast cancer cells and MCF-12A noncancerous epithelial breast cells using the MTT assay [12]. In this study, we used the xCELLigence RTCA system (ACEA Biosciences, Roche, Germany) to conduct a detailed investigation of the effect of vulpinic acid on MCF-7 and MCF-12A in real time. The xCELLigence RTCA system was continuously monitored for cytotoxicity and was based on electronic sensor array technology. Each type of cell was seeded at 1 × 10^4^ cells/well in E-plate 16 (ACEA Biosciences, RTCA, Roche, Germany). Controls were treated with a culture medium containing 0.05% (v/v) DMSO. Each well with 100 µL medium was seeded and impedance was recorded at 15 min intervals. After 24 h, various concentrations of vulpinic acid (1.56, 3.125, 6.25, 12.5, 25, 50 and 100 µM) were added to the well/E-plate and monitored every 15 min at 37° C in a 5% CO_2_ atmosphere. The Normalized Cell Index (NCI) is the manipulation of data as 1.0 (100% values) by the software of xCELLigence RTCA system where a specific time is chosen. Each value measured in the cell is proportioned according to NCI. Thus, the adhesion rate (%) of cells to the e-plate surface is determined by NCI. The cell index (CI) graph and IC_50_ values were obtained using the xCELLigence RTCA software program (RTCA, ACEA Biosciences, Roche, Germany).

### Total RNA extraction

MCF-7 and MCF-12A cells were seeded at a density of 5 × 10^5^ cells in 6 well plates, and then the cells were treated with IC_50_ concentration of vulpinic acid for 48 h. Total RNA and miRNAs were extracted using the TRIzol reagent according to the manufacturer’s protocol. The quantity of total RNA was determined using the Nanodrop ND-1000 Spectrophotometer (Thermoscientific, USA) and showed using 1% agarose gel electrophoresis. The quality of RNA samples was controlled using the Bioanalyzer (Bioanalyzer 2100, Agilent Technologies, Santa Clara, CA).

### miRNA expression profile assay

A total of 500 ng RNA was hybridized using the Human miRNA Microarray (Agilent Technologies, Santa Clara, CA, USA). The expression analyses of vulpinic acid responsive miRNAs were performed according to Agilent’s protocol. Ligation of the Cyanine 3-pCp molecule to the 3′ end of RNAs resulted in labeling the total RNA obtained from each cell. Then, oligoarrays were hybridized with fluorescently labelled targets for 20 h in a hybridization oven. After the hybridization stage, buffers provided by the manufacturer (Agilent Technologies) were used on slides to eliminate the nonspecific hybridization. Then, the drying process was performed by washing the slides in acetonitrile for 1 min and in a washing and stabilization solution for an additional 1 min. The arrays were transformed digitally using a scanner and software (Agilent Technologies, Santa Clara, CA, USA).

### Data analysis from the miRNA array data

BRB-Array Tools were applied for the array analysis. Normalization of the raw microarray data was performed using the quantile normalization method. Then, the results were filtered for miRNAs to determine whether less than 20% of their expression data had at least a twofold change in either direction from the median value of the miRNA. Differently used normalization methods validated the microarray data for the effects of vulpinic acid. The microarray assays were also evaluated using the Banforrine test (p ˂ 0.05, FC ˃ 2). Another statistical method, the class comparison test, was used to calculate the significance (p ≤ 0.05, twofold change) of differently expressed miRNAs among the samples (vulpinic acid and DMSO-treated MCF-7 and MCF-12A). At the end of the analysis, differently expressed miRNAs were identified and classified using the bioinformatics tool and Venn diagrams (Venny 2.1.0).

### miRNA target prediction and pathway analysis

The target genes of differentially expressed vulpinic acid responsive miRNAs were analyzed using the DIANA (http://diana.imis.athena-innovation.gr/DianaTools/index.php), TargetScan (http://www.targetscan.org/) and mirDB (http://www.mirdb.org/) tools to determine miRNA targets and further pathway analysis.

Moreover, miRwalk3, an experimentally validated miRNA target interactions database, was used to predict the target genes of the 12 differently expressed miRNAs. The candidate genes of vulpinic acid responsive miRNAs were determined using Gene Ontology (GO) and KEGG pathways based on their functional roles and most related pathways.

### Validation of miRNAs using qRT-PCR

The differential miRNA expression patterns were validated through qRT-PCR assay using the miRCURY LNA™ Universal RT microRNA PCR (Exiqon, Germany) according to the manufacturer’s protocol. The qRT-PCR reactions were performed as denaturation at 95° C for 10 min, 45 amplification cycles at 95 °C for 10 s and 60 °C for 1 min using the Light Cycler 480 thermocycler (Roche, Germany). RNU6 was used as the endogenous control for qRT-PCR of miRNAs. Each sample was run in duplicate, and the relative quantification of the miRNA expression level was determined using the ΔΔCt method [28].

### qRT-PCR analysis of the target genes

The target genes of differentially expressed vulpinic acid responsive miRNAs were confirmed using quantitative real-time RT-PCR analysis. All samples were examined in triplicate, and the expression of each gene was standardized using the housekeeping gene glyceraldehyde-3-phosphate dehydrogenase (GAPDH) as a reference gene. Amplification reactions were performed using the Light Cycler 480 real-time PCR instrument (Roche, Germany). The conditions for the reactions were as follows: 95 °C, 10 min; 95 °C, 15 s; and 60 °C, 60 s for 40 cycles. The expression of related genes was determined using the 2^△△ct^ method [28].

### Western blotting

All proteins were isolated in a lysis buffer (Cell Signaling Technology, MA, USA) supplemented with protease and phosphatase inhibitors (Cell Signaling, MA, USA). MCF-7 breast cancer cells were treated with 18 µM vulpinic acid (IC_50_ concentration), to which was added about 200 µl lysis buffer, and incubated for 30 min. The lysates were kept on ice at all steps and were clarified by centrifugation at 14,000 g for 10 min at 4 °C. The lysates were boiled for 5 min in the presence of a loading buffer with a dry block heater (Major Science, USA). A quantity of 30 μg proteins was resolved on a 12% sodium dodecyl sulfate (SDS)-polyacrylamide gel (PAGE) by electrophoresis at 100 V for 90 min. The resolved proteins were transferred to polyvinylidene difluoride (PVDF) membranes (Thermo Fisher Scientific, USA) using the semi-dry transfer system (Cleaver, UK). The membranes were then incubated with a blocking buffer (5% w/v nonfat dry milk) in a Tris-buffered saline containing 0.1% v/v Tween-20 (TBST) for 2 h at 37 °C and incubated overnight at 4 °C with FOXO-3, Bcl-2, Bax, procaspase-3 and procaspase-9 antibodies (1:2000) (Cell Signaling Technology, MA, USA) in a blocking buffer and mouse anti-β-actin secondary antibodies (1:1000) (Cell Signaling Technology, MA, USA) in phosphate-buffered saline (PBS), which was used as an internal control. The membranes were washed four times with TBST for 10 min per wash at room temperature, followed by incubation with the horseradish peroxidase (HRP)-conjugated anti-mouse IgG antibody (1: 2000) in PBS (Cell Signaling Technology, USA) for 90 min. The membranes were washed four times with TBST, 10 min per wash, at room temperature. Finally, the membranes were exposed to a chemiluminescence reagent (SuperSignal West Pico Chemiluminescent Substrate, Thermo Fisher Scientific, USA) according to the manufacturer’s instructions. Images of the western blots were visualized using the Licor Imaging System (LI-COR, Odyssey Fc 2800, USA). The relative expression ratio of all the examined antibodies was calculated as follows: relative expression ratio of primer antibody integrated optical density (IOD)/β-actin IOD.

### Statistical analysis

A Student’s t-test was used to compare the two groups. The *p-*value was set at 0.05 to determine the significant differences.

## Results

### Online monitoring of cytotoxic activity using the xCELLigence RTCA instrument

**Fig. 1:**
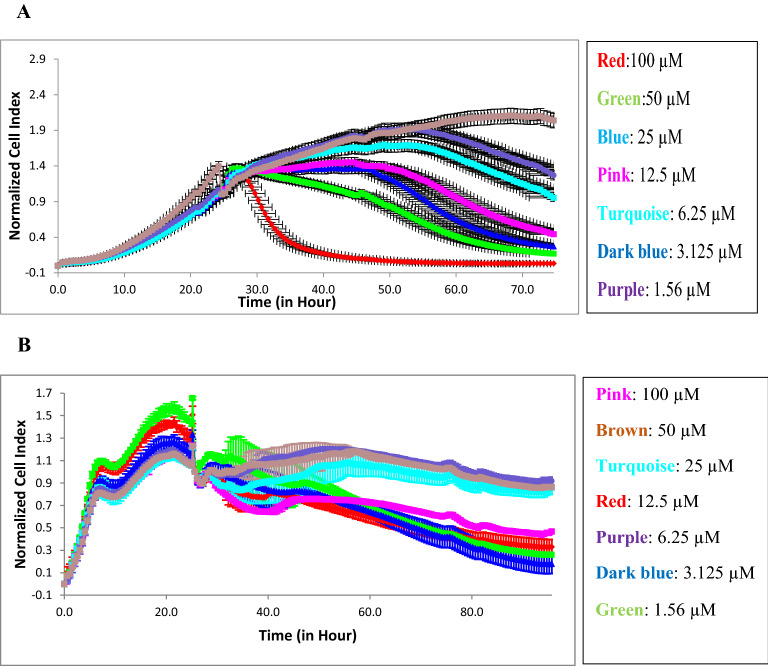
Cell index of vulpinic acid seeded at different concentrations monitored over 96 h on MCF-7 (**A**) and MCF-12A (**B**) cells. Data represent mean ± SEM (n = 3). Average change of cell index of vulpinic acid exposed MCF-7 cells from non-treated cells over 96 h. (NCI: Normalized Cell Index)

Experiments were carried out using the xCELLigence RTCA system to determinate the antiproliferative effect of vulpinic acid on MCF-7 breast cancer cells and MCF-12A noncancerous epithelial cells. Seven concentrations of vulpinic acid were incubated with cells for 96 h, and the plates were monitored every 15 min. The IC_50_ value was calculated on the basis of CI at every measuring point of the experiment. Figure 1A and B represent the concentration-dependent antiproliferative effect of vulpinic acid on MCF-7 and MCF-12A cells. Vulpinic acid caused cytotoxicity for MCF-7 cells at 100 µM. The dose of 1.56 µM seems to have a cytostatic effect on MCF-7 cells. The IC_50_ value of vulpinic acid obtained using the RTCA system was 18.0 ± 0.02 μM for MCF-7 breast cancer cells based on the sigmoidal dose–response formula (Fig. 1A). A control sample (DMSO 0.5% [v/v]) did not inhibit the cell growth. Additionally, we prepared the same experiments using the MCF-12A noncancerous epithelial breast cells (Fig. 1B). Using the xCELLigence RTCA system, vulpinic acid was shown to have an antiproliferative effect on MCF-7 cells, but had little effect on MCF-12A noncancerous epithelial breast cells. The IC_50_ value of vulpinic acid in MCF-12A epithelial cells was found to be 6.2 μM after a 96 h incubation period. The obtained results indicate that MCF-7 breast cancer cells were sensitive to vulpinic acid, and the viability of MCF-7 breast cancer cells significantly decreased after treatment with the vulpinic acid. These findings clearly indicate that vulpinic acid has a potent anti-growth effect on MCF-7 breast cancer cells. In this regard, vulpinic acid is a kind of natural anticancer candidate molecule for breast cancer that is nontoxic and easily accessible in nature.

### Microarray data

**Fig. 2:**
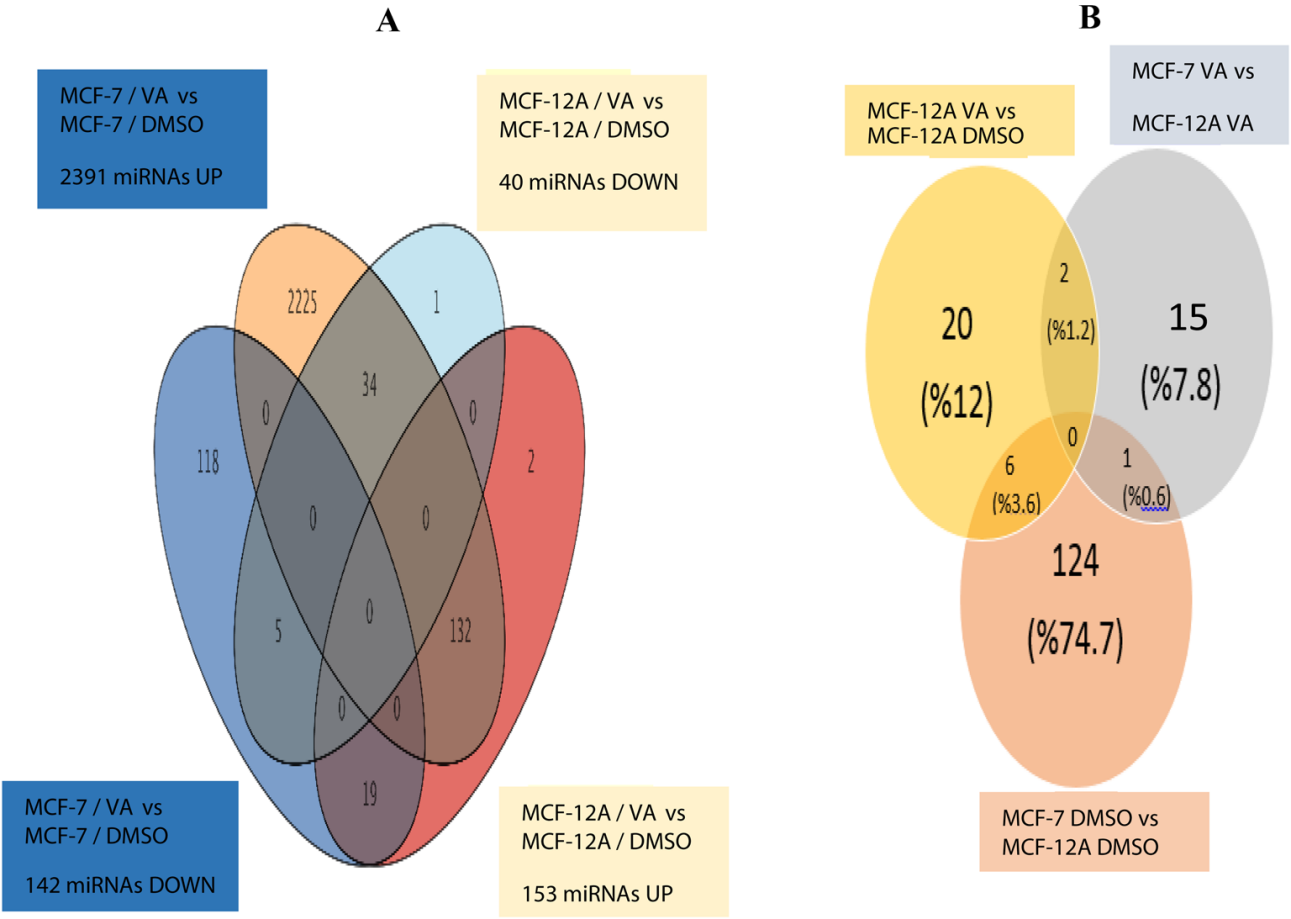
miRNA expression profile induced by vulpinic acid. **A** Venn diagram representation of miRNA expression profile with comparison of MCF-7 VA-MCF-7 DMSO vs MCF-12 VA-MCF-12A DMSO; **B** the filtered status of miRNA expression analysis results according to Bonferroni FWER method and p < 0.05

Vulpinic acid exerts anticancer effects on MCF-7 breast cancer cells. However, the correlation between vulpinic acid and miRNA remains unknown. The cells (MCF-7 and MCF-12A) were treated with IC_50_ values of vulpinic acid for a microarray experiment. RNA was isolated from MCF-7 and MCF-12A cells treated with vulpinic acid. The purification of RNA was determined using gel electrophoresis and an electropherogram of MCF-7 and MCF-12A cell lines treated with vulpinic acid (Additional file 1: Fig. S1). The miRNA array data were normalized, and the results are given in Additional file 1: Fig. S2. We distinguished four different categories of MCF-7-specific miRNAs and MCF-12A-specific miRNAs: the first category, *MCF-7/VA* vs *MCF-7/DMSO*, with an *increased* expression of miRNAs; the second category, *MCF-7/VA* vs *MCF-7/DMSO*, with a *decreased* expression of miRNAs; the third category, *MCF-12A/VA* vs *MCF-12A/DMSO*, with an *increased* expression of miRNAs; and the fourth category, *MCF-12A/VA* vs *MCF-12A/DMSO*, with a *decreased* expression of miRNAs. These four different categories of miRNA data were compared with the Venn diagram (Fig. 2A). In the MCF-7 cells treated with vulpinic acid and the untreated cells, 2533 specific miRNAs were identified. Among the 2533 different miRNAs, 2391 were significantly upregulated and 142 were downregulated. Similarly, 193 miRNAs were detected in MCF-12A noncancerous breast epithelial cells. In the MCF-12A cells, 40 miRNAs were upregulated and 153 miRNAs were downregulated (Fig. 2A).

**Fig. 3:**
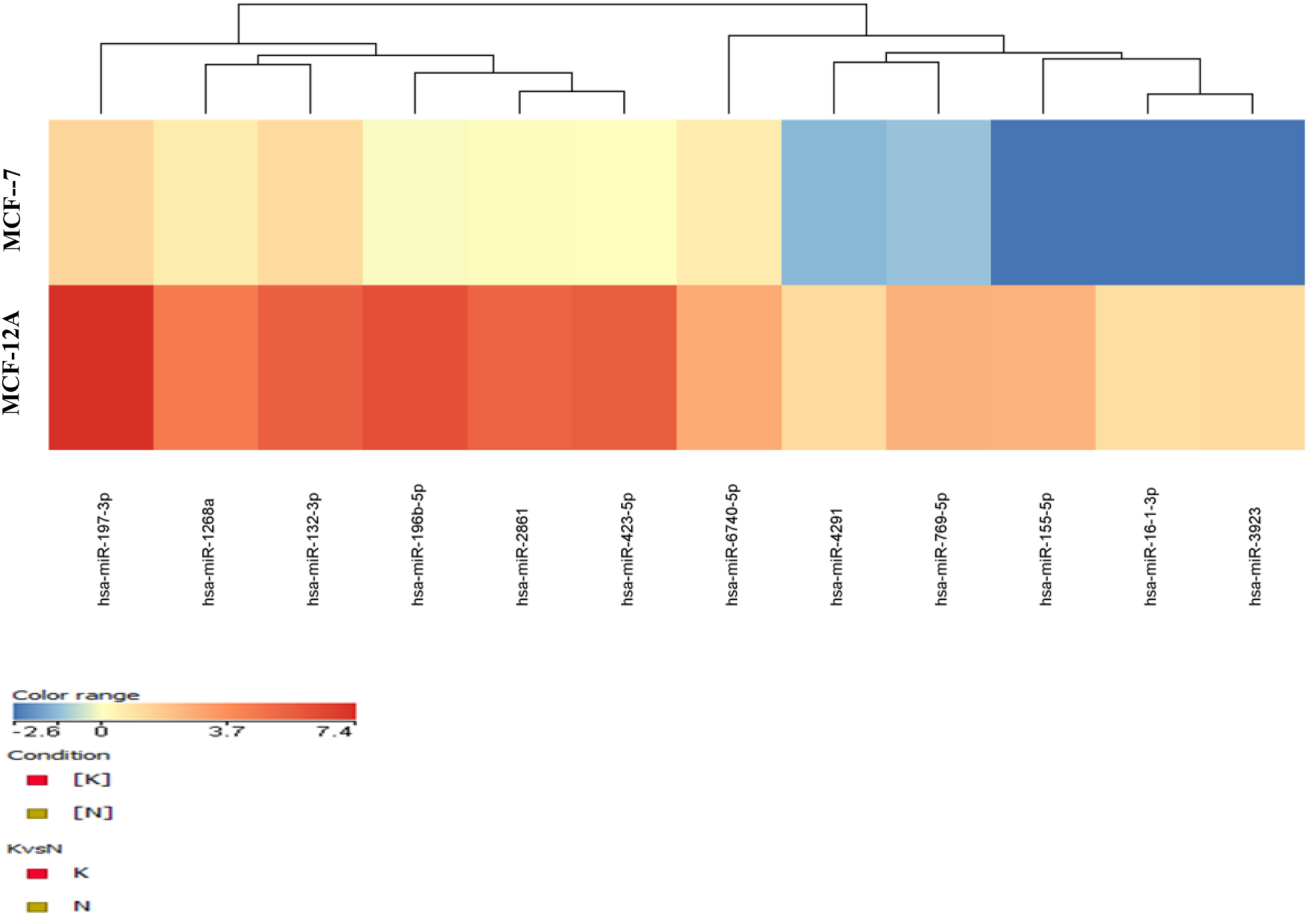
Heat map of differentially expressed miRNAs by vulpinic acid in MCF-7 breast cancer cells

For further analysis, all of the related filter methods (the Bonferroni FWER analysis method, fold change ˃2 and p < 0.05 method) were applied to the obtained results and presented in the Venn diagram (Fig. 2B). The aim in the application was to identify MCF-7-specific miRNAs other than MCF-12A-specific miRNAs and drug solvent (DMSO) through statistical analysis. Following the statistical analysis, 175 miRNAs were identified for all categories. Among the 175 miRNAs, 16 were categorized as MCF-7 vs MCF-12 cells following vulpinic acid application, while 28 were identified as MCF-12A/VA vs MCF-12A/DMSO and 131 were identified as MCF-7/DMSO vs MCF-12A/DMSO (Fig. 2B). When the common intersection of these categories was observed in the Venn diagram, 12 miRNAs (miR-1268a, miR-132-3p, miR-155-5p, miR-16–1-3p, miR-196b-5p, miR-197-3p, miR-2861, miR-3923, miR-423-5p, miR-4291, miR-6740-5p and miR-769-5p) were found at the MCF-7-specific significance level (Fig. 2B). In Fig. 3, the heat map shows specific statistically significant miRNAs generated in response to the treatment of MCF-7 cells with vulpinic acid.

### Validation of the expression levels of vulpinic acid responsive miRNAs through qRT-PCR assay

**Fig. 4:**
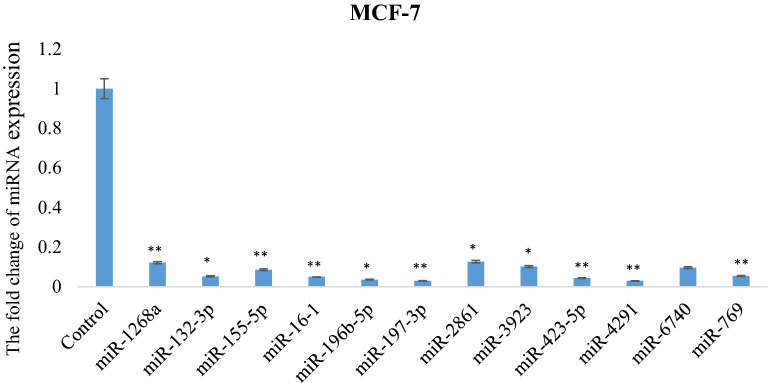
The validation of miRNA array data by quantitative real-time PCR analysis. The mean Ct values of vulpinic acid responsive 12 miRNAs were determined in MCF-7 breast cancer cells treated with IC_50_ (18 µM) concentrations of vulpinic acid for 48 h. RNU6 was used as control gene (*p < 0.05, **p < 0.01)

The 12 statistically valuable expressed miRNAs obtained from the microarray data were validated using a qRT-PCR assay (Fig. 4). The expression level of miR-1268a showed about an eightfold decrease in MCF-7 cells (p < 0.01). The expression level of miR-132-3p showed a greater decrease (19.16-fold) in MCF-7 cells compared with miR-1268a (p < 0.05). In the expression analysis of MCF-7cells treated with vulpinic acid, there was a significant decrease in the expression level of miR-155-5p (11.71-fold) (p < 0.01). The expression level of miR-16–1-3p statistically decreased (19.83-fold) in MCF-7 (p < 0.01). The expression level of miR-196-5p showed an approximately 30-fold decrease (p < 0.05). The expression level of miR-197-3p decreased by 33.59-fold in MCF-7 cells (p < 0.01). The expression level of miR-2861 and miR-3923 showed similar changes (about tenfold) in MCF-7 cells (p < 0.05). The result of the miR-423-5p expression level demonstrated a significant 22.78-fold decrease (p < 0.01). The results indicated that miR-4291, miR-6740-5p and miR-769-5p increased in MCF-7 cells by a 34.05- (p < 0.01), 10.41- and 18.5- (p < 0.01) fold change, respectively (Fig. 4). As a result of the microarray analysis, the expression level of the 12 miRNAs decreased, which was confirmed by the qRT-PCR assay.

### Bioinformatic analysis

**Fig. 5:**
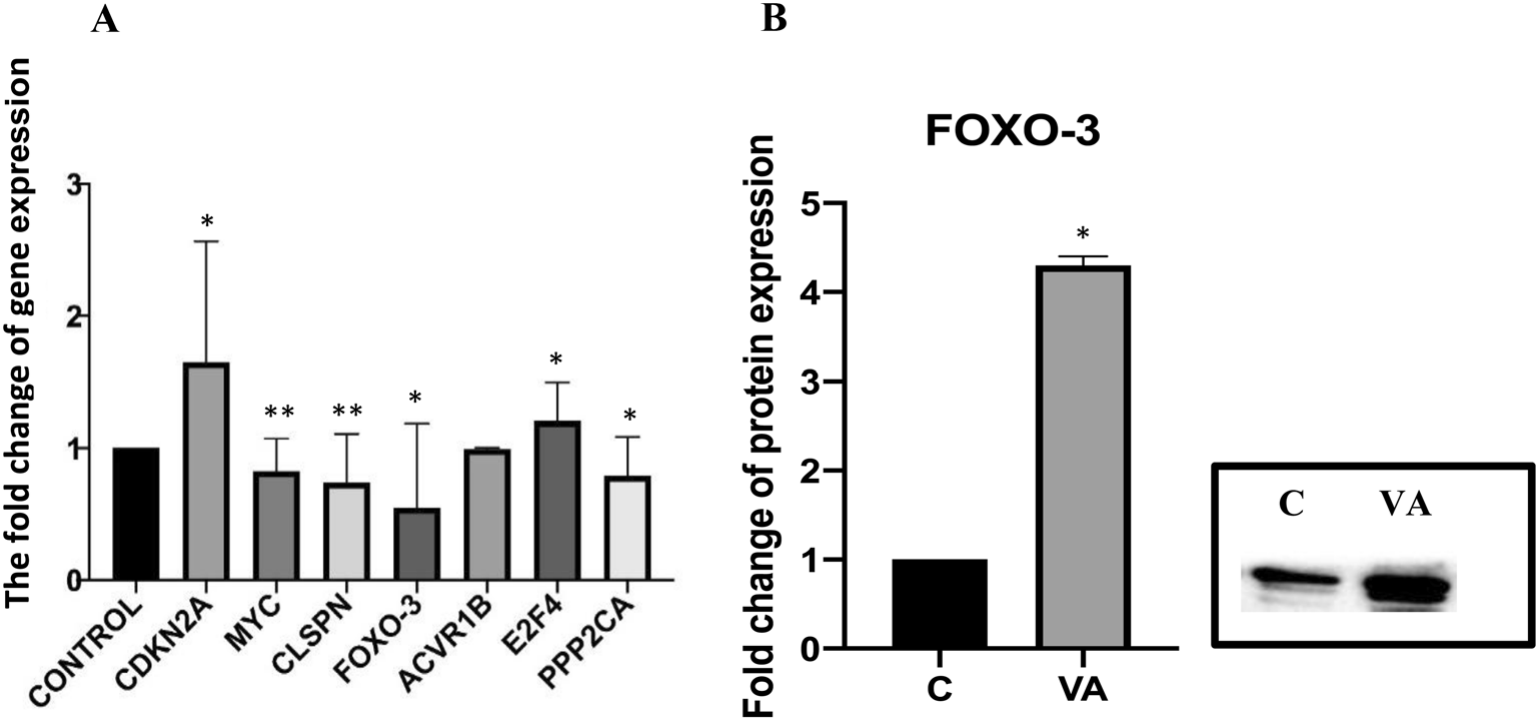
The expression level of *CDKN2A, MYC, CLSPN, FOXO3, ACVR1B, E2F4* and *PPP2CA* genes of differentially expressed vulpinic acid responsive-miRNAs (**A**); The effect of vulpinic acid on protein expression of FOXO3 in MCF-7 breast cancer cells (**B**) (*p < 0.05, **p < 0.01)

We used three bioinformatics tools, TargetScanHuman, miRDB and DIANA, to determine the biological functions and target genes of the 12 differently expressed miRNAs (Table 1). In the bioinformatic analysis of the 12 statistically significant miRNAs that respond to vulpinic acid, 7 target genes (*CDKN2A, MYC, CLSPN, FOXO-3, ACVRIB, E2F4* and *PPP2CA*) common to the 12 miRNAs were identified. These target genes play significant roles in the apoptosis, cell cycle and cell proliferation pathways. We used a qRT-PCR assay to validate the regulatory effects of vulpinic acid as a candidate natural compound for targeting miRNA. Among the genes that were significantly altered when treated with vulpinic acid in MCF-7 cells, four downregulated genes (*MYC, CLSPN, FOXO-3* and *PPP2CA*) and three upregulated genes (*CDKN2A, ACVR1B* and *E2F4*) were validated through quantitative real-time RT-PCR. The same RNA samples used for microarray were used to generate cDNA templates for reverse transcription reactions. Consistent with the microarray data, the 7 selected genes showed a similar expression profile as presented in the microarray data (Fig. 4). In the current study, we determined for the first time that vulpinic acid may downregulate miR-1268a, miR-132-3p, miR-155-5p, miR-16–1-3p, miR-196b-5p, miR-197-3p, miR-2861, miR-3923, miR-423-5p, miR-4291, miR-6740-5p and miR-769-5p and inhibit cell proliferation in MCF-7 cells. The downexpression of these miRNAs significantly reduced cancer cell proliferation and apoptosis by targeting *FOXO-3, CLSPN, ACVR1B, E2F4* and *PPP2CA* and regulated cell cycle progression by targeting *CDKN2A* and *FOXO-3* (Fig. 5A).

**Table 1:** The number of gene targets of 12 miRNAs deregulated by vulpinic acid in MCF-7 breast cancer cells

miRNA	Primer sequences	TargetScanHuman	miRDB	DIANA
miR-1268a	5′-CGGGCGUGGUGGUGGGGG-3′	1790	83	25
miR-132-3p	5′-UAACAGUCUACAGCCAUGGUCG-3′	474	673	1379
miR-155-5p	5′-UUAAUGCUAAUCGUGAUAGGGGU-3′	556	701	5894
miR-16–1-3p	5′-CGAAUCAUUAUUUGCUGCUCUA-3′	3917	735	311
miR-196b-5p	5′-UAGGUAGUUUCCUGUUGUUGGG-3′	374	369	814
miR-197-3p	5′-UUCACCACCUUCUCCACCCAGC-3′	4888	465	625
miR-2861	5′-GGGGCCUGGCGGUGGGCGG-3′	4407	524	0
miR-3923	5′-AACUAGUAAUGUUGGAUUAGGG-3′	1926	215	15
miR-423-5p	5′-UGAGGGGCAGAGAGCGAGACUUU-3′	232	700	1112
miR-4291	5′-UUCAGCAGGAACAGCU-3′	5096	1403	0
miR-6740-5p	5′-AGUUUGGGAUGGAGAGAGGAGA-3′	3798	443	0
miR-769-5p	5′-UGAGACCUCUGGGUUCUGAGCU-3′	3669	296	326

In addition, further analysis of the related pathways of the 12 specific miRNAs was conducted using the KEGG, GOBP, WikiPath and PanterDB tools (Table 2). The pathway analysis using the four different bioinformatics tools did not detect related pathways for miR-2861, miR-3923, miR-4291 and miR-6740-5p (Table 2). Predicted targets common to two or more databases were selected for further analysis (Table 2), and the genes were found to be associated with cancer, cell viability, cell cycle, P53 signaling pathway, apoptosis, MAPK, Wnt and JAK/STAT signaling pathways. In this study, miRNA-targeting vulpinic acid was identified as equally or more effective as other chemotherapeutics. Among these 12 miRNAs, miR-197-3p and miR-423-5p were relatively less studied in the context of tumor formation and progression in breast cancer. In order to test their potential role as tumor suppressors, we performed a viability assay on breast cancer cell lines representing different clinical subtypes. We observed that miR-197-3p and miR-423-5p had a growth inhibitory effect and thus decided to focus on these miRNAs for further study.

**Table 2:** Cellular pathways modulated by 12 differentially expressed miRNAs in MCF-7 cells treated with vulpinic acid

MiRNA	KEGG	GOBP	WikiPath	PanterDB	Common pathways
miR-1268a	8	24	3	–	•Apoptosis pathways •MAPK signaling pathways •PI3K/Akt signaling pathways
miR-132-3p	172	1227	137	61	
miR-155-5p	877	5192	534	357	
miR-16–1-3p	20	37	12	3	
miR-196b-5p	75	425	41	31	
miR-197-3p	166	1021	103	75	
miR-2861	–	–	–	–	
miR-3923	–	–	–	–	
miR-423-5p	177	1172	128	92	
miR-4291	–	–	–	–	
miR-6740-5p	–	–	–	–	
miR-769-5p	137	511	83	45	

### Western blotting analysis

*FOXO-3* plays significant roles in biological processes, including cancer, and is actively involved in promoting apoptosis in a mitochondria-independent and -dependent manner. In this study, 7 common target genes for 12 miRNAs that respond to vulpinic acid were determined. Among these 7 target genes, *FOXO-3* has the highest expression level. Since the expression level of the 12 miRNAs decreased at the miRNA level as a result of vulpinic acid application, *FOXO-3* was selected for protein-level verification in this study. Vulpinic acid inhibited the 12 miRNAs and MCF-7 breast cancer cell proliferation, promoted Mcf-7 breast cancer cell apoptosis, decreased the expression of the FOXO-3 gene and FOXO-3 protein (Fig. 5B).

**Fig. 6:**
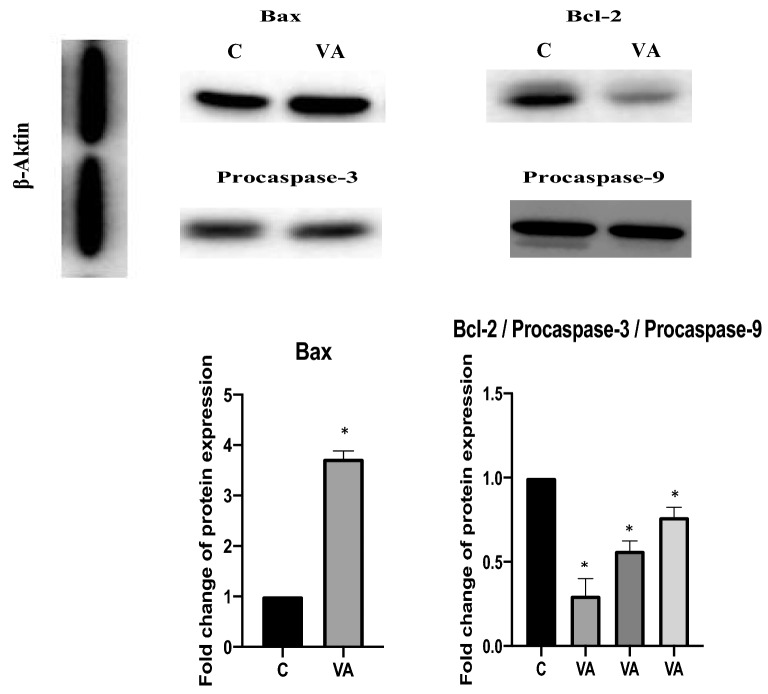
The expression level showed that Bax, Bcl-2, procaspases-3 and procaspases-9 proteins as measured by western blot (*p* < 0.001*). The ratio of Bax significantly increased with apply to vulpinic acid (*p* < 0.001*) and the protein levels of Bcl-2, procaspases-3 and procaspases-9 decreased significantly with vulpinic acid

Western blotting analysis was performed to detect the changes in the levels of proteins associated with the apoptotic signaling pathway. In this study, we showed an increase in Bax protein levels in MCF-7 cells treated with vulpinic acid and detected decreased cleaved Bcl-2, procaspase-3 and procaspase-9 in MCF-7 cells (Fig. 6). The results indicate that vulpinic acid increased proapoptotic protein expression and decreased antiapoptotic protein expression via the targets of the 12 miRNAs. In summary, vulpinic acid has a tumor-suppressing function in MCF-7 cells.

## Discussion

Cancer treatment options such as surgery, chemotherapy and monoclonal antibodies have been applied for breast cancer, but these options are not significantly effective due to their side effects and the advent of chemoresistance. In this situation, novel and alternative molecules are necessary for the treatment of breast cancer. Natural products have been a main source of drug discovery for many decades. It is estimated that more than half of all cancer drugs have originated from a chemical compound discovered in a natural product. One method of searching for potential anticancer drugs is to test various naturally synthesized compounds. Plant samples are also the largest provider of marketed anticancer drugs, including paclitaxel (Taxol) and docetaxel. Paclitaxel is one of the most commonly used chemotherapy drugs for ovarian, breast and lung cancer and is derived from the bark of the Pacific yew tree. The resistance to chemotherapeutics occurs during the treatment of cancer, and this stage is a significant obstacle for future use. Lichens are a source of unique biological agents that have already been proven to be effective against various cancers. Lichen secondary metabolites have gained increasing importance for use in cancer treatment to inhibit cell proliferation and induce apoptosis of the different cancer cells. Vulpinic acid, one of the small molecule metabolites derived from lichens (lichenized fungi), has been shown to have pharmacological effects such as anticancer effects on different cancer cells. However, vulpinic acid has not shown any significant cytotoxic effects on the viability of MCF-12A cells. Small molecules derived from lichens are a very important resource for promising compounds in oncology.

Many studies suggest that targeting miRNAs using natural products is a new therapeutic option for cancer therapy. These miRNAs could serve as cancer biomarkers for diagnosis, prognosis and therapeutic targets. Anticancer drugs such as paclitaxel, resveratrol and curcumin are derived from natural products and have antiproliferative and apoptotic effects through the regulation of miRNAs. In this study, we determined the response of miRNAs in MCF-7 breast cancer cells and MCF-12A noncancerous epithelial cells to vulpinic acid. The data obtained from this study will help us better understand how vulpinic acid regulates miRNAs, determine therapeutic targets and potentially consider vulpinic acid as a promising alternative anticancer compound for breast cancer treatment. This is the first study related to the description of vulpinic acid, a lichen secondary metabolite, in breast cancer at miRNA level. This work will enable the complete eradication of tumors by killing drug-resistant cells to improve survival outcomes in patients diagnosed with malignancy.

**Fig. 7:**
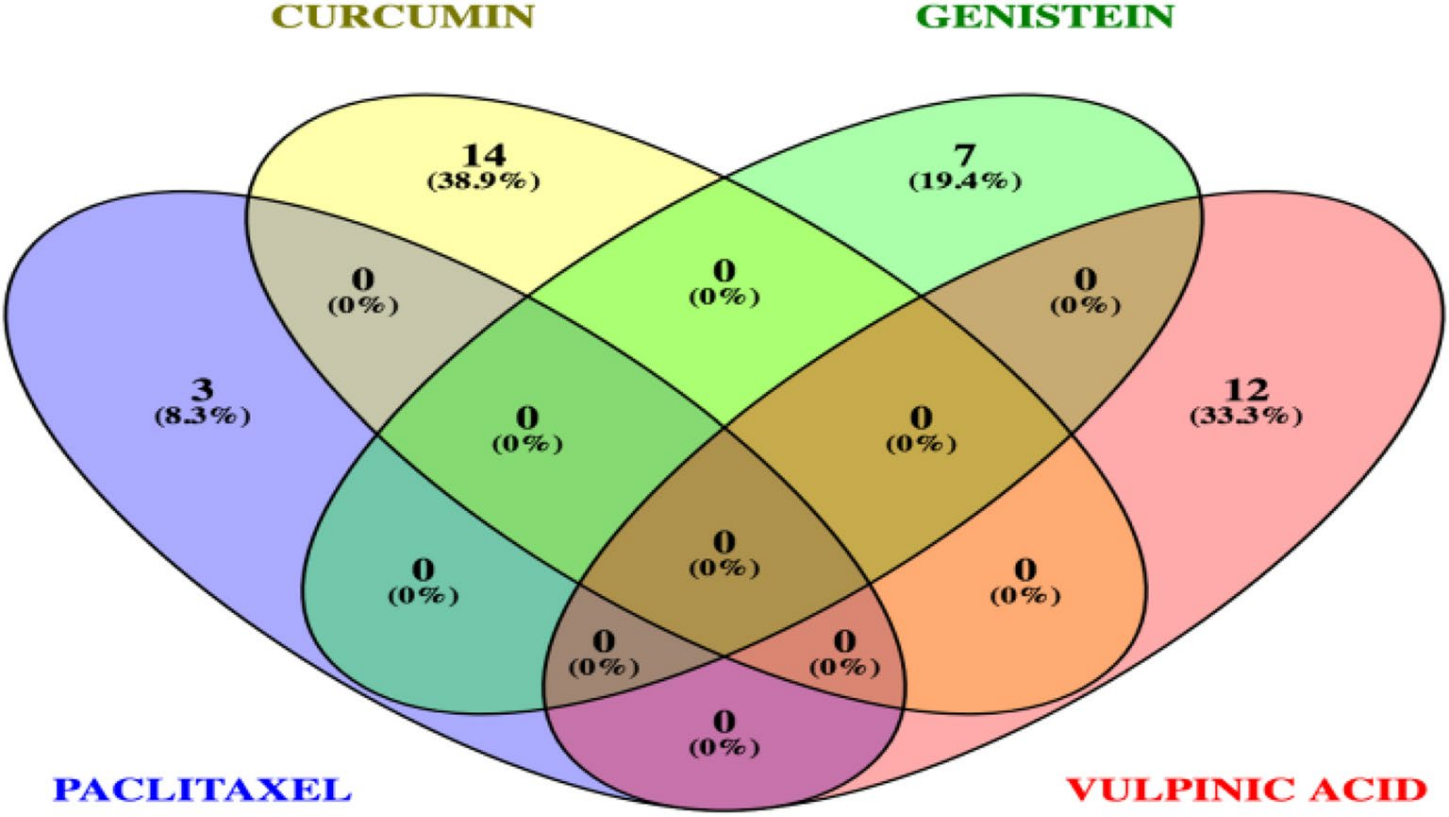
The comparison of miRNAs of chemotherapeutics such as paclitaxel, curcumin and genistein and vulpinic acid response miRNAs

In the literature, it has been demonstrated that miRNAs could be used as therapeutic targets and biomarkers for cancer. Roberts et al. (2013) determined that curcumin inhibits cell proliferation and induces the cell cycle and apoptosis in different cancer cell lines [29]. Norouzi et al. (2018) have also reported curcumin-related miRNAs in breast cancer [30]. As a result of the study of Norouzi et al., the upregulation of miR-181b, miR-34a, miR-16, miR-15a and miR-146b-5p and downregulation of miR-19a and miR-19b have been found in the treatment of different breast cancer cells with curcumin [20, 30]. Downregulated miRNAs have been identified for various natural products in different studies [20]. As a result of these studies, downregulated miR-107 has been shown for paclitaxel [31–33]; downregulated miR-199a, miR-1246, miR-7641, miR-19a and miR-19b have been shown for curcumin [30, 34]; and downregulated miR-155 has been shown for genistein [35]. Otsuka et al. (2018) found that [20, 36] genistein as a natural compound increases the expression of miR-451 in cardiac hypertrophy [36]. Ma et al. (2018) showed that genistein increases miR-1469 by inhibiting Mcl-1 expression in laryngeal cancer [37]. In another study, Dong Wei et al. (2017) determined that miR-145 upregulated after applied to genistein by antiproliferative effect on retinoblastoma cell and induced apoptosis [38]. Kılıç et al. (2019) determined that 67 miRNAs are specifically responsive to usnic acid (lichen secondary metabolite) in MDA-MB-231 cells, while 15 and 8 are specific to BT-474 and MCF-7 cells, respectively [27]. Değerli et al. (2020) identified miR-185-5p as the most promising usnic acid response of miRNAs in BT-474 breast cancer cells [39]. In this study, we determined that vulpinic acid responsive miRNAs. However, we found that miRNAs responsive to vulpinic acid differ from miRNAs responsive to chemotherapeutics such as paclitaxel, curcumin and genistein (Fig. 7) [20]. Similarly, a common miRNA responsive to usnic acid and vulpinic acid has not been identified. These data reveal that vulpinic acid could modulate the expression of specific miRNAs, and this is an important sign for the potential treatment of breast cancer in the future.

Misir et al. (2020) observed that propolis has been shown to regulate apoptosis signaling pathways and alter the expression of miRNAs [40]. Ethanolic propolis extract elevated apoptotic cell death by increasing proapoptotic protein levels (p21, Bax, p53, p53-Ser46 and p53-Ser15), decreasing mitochondrial membrane potential and altering the expression levels of specific tumor suppressors (miR-34, miR-15a and miR-16-5p) and oncogenic (miR-21) miRNAs. These data support the argument that Turkish propolis may be evaluated as a potential natural agent for new anticancer drugs [40]. Similarly, the present study reports that the vulpinic acid regulated apoptosis pathway by 12 miRNAs and vulpinic acid as an miRNA-targeted natural product might be potentially used in breast cancer treatment.

Zhang et al. (2018) found that the miR-433 expression levels were significantly reduced in breast cancer and that the overexpression of miR-433 inhibited cell proliferation and migration and induced apoptosis [41]. In their study, the candidate target gene *Rap1a* was determined as a target of miR-433. *Rap1a* has been linked to cell division, proliferation, apoptosis and the differentiation of the stages of cancer. In the present study, we examined the effects of vulpinic acid on miRNA levels of MCF-7 breast cancer cells and its relationship with cell proliferation and apoptosis. *FOXO-3* genes were determined as a common target of 12 miRNAs that respond to vulpinic acid. *FOXO-3* genes act as tumor suppressors by promoting cell cycle arrest and apoptosis [42]. Vulpinic acid induces *FOXO-3* in MCF-7 cells and apoptosis activated with trigger intrinsic and extrinsic pathways targets through 12 miRNAs expression.

Carcinogenesis is related to an imbalance between cell proliferation and apoptosis stage. Genes that control the cell cycle and apoptosis have the potential to impact drug sensitivity and resistance [43]. Next-generation sequencing revealed the role of epigallocatechin gallate (EGCG) in regulating MAPK signaling by targeting several miRNAs in non-small cell lung cancer A549 cells [44]. EGCG has also been found to attenuate uric acid-induced injury in NRK-49 F cells by upregulating miR-9 and subsequently activating NF-κB and JAK-STAT signal pathways [45]. 6-Gingerols reduced hypoxia-caused apoptosis and autophagy in PC-12 cells by regulating miR-103/BNIP3 axis [46]. Another study demonstrated that downregulation of miR-210 was involved in 1′S-1′-acetoxychavicol acetate (ACA)-induced cervical cancer cell apoptosis by targeting SMAD4 [47]. The different mechanisms underlying the anticancer effects of lichen secondary metabolites may include downregulated gene expression, leading to growth inhibition, inducing apoptosis and regulating various signaling pathways. In regard to cancer-associated molecular mechanisms such as programmed cell death, lichens act as activators of apoptosis in various cancer cells [8, 48]. Induction of apoptosis by lichens might also be associated with an increase of cleaved PARP, a stress-response protein repairing damaged DNA and regulating chromatin structure [49], and inactivation of the mammalian target of rapamycin (mTOR) or activation of c-Jun N-terminal kinase (JNK) signaling [50]. The antiproliferative effects of lichens can be modulated through the regulation of other signaling pathways such as ERK1/2, MAPK and AKT [51] or the proliferation protein marker Ki-67. In the current study, vulpinic acid was found to downregulate the expression of 12 miRNAs by targeting mainly genes related to apoptosis and cell cycle, and these vulpinic acid regulated miRNAs were important to the anticancer effect of vulpinic acid. Tamoxifen treatment induces cell cycle arrest via the regulation of cyclin D1, RB1 and CDKN1A and induces apoptosis by decreasing Bcl-2 and increasing Bax expression [43]. In this study, we determined that vulpinic acid treatment induces apoptosis by regulating *FOXO-3*, decreasing Bcl-2, pro-caspase-3 and pro-caspase-9 and increasing Bax expression. In this sense, tamoxifen and vulpinic acid may have a similar mechanism of action and common miRNA response. The experimental evidence shows that vulpinic acid may be a candidate molecule that can be used in breast cancer treatment, much like tamoxifen. However, no common miRNAs were identified among the 12 miRNAs that respond to vulpinic acid and the miRNAs that respond to paclitaxel, curcumin and genistein. These data support the argument that vulpinic acid may be evaluated as a potential natural agent for new anticancer drugs in breast cancer compared with other chemotherapeutics. A very limited number of studies have explored the molecular targets and/or signaling pathways through which lichen-derived vulpinic acid metabolites exert their anticancer activity with the targeting of miRNA. Our results demonstrate the anticancer activity of vulpinic acid and the 12 vulpinic acid related miRNAs mediated via the induction of apoptosis.

## Conclusion

This study was based on an in vitro model, and its results provide novel insights into the molecular characterization of the effect of vulpinic acid on breast cancer at the miRNA level. Vulpinic acid elevated apoptotic cell death by increasing proapoptotic protein levels (FOXO-3, Bax), decreasing Bcl-2, pro-caspase-3 and pro-caspase-9 and altering the expression levels of specific tumor suppressor miRNAs (miR-1268a, miR-132-3p, miR-155-5p, miR-16–1-3p, miR-196b-5p, miR-197-3p, miR-2861, miR-3923, miR-423-5p, miR-4291, miR-6740-5p and miR-769-5p). There are a number of in vitro and in vivo studies demonstrating the anticancer effects of lichen secondary metabolites, but no data related to the vulpinic acid response of miRNA has yet been generated. The investigation of the vulpinic acid response of miRNAs and determination of pathways are important steps towards the development of more effective treatment strategies through precision medicine. These novel findings suggest that the use of vulpinic acid as a natural agent could open new avenues for the successful treatment of breast cancer, and vulpinic acid may be evaluated as a potential natural agent for new anticancer drugs in the future. However, in vivo studies are required to confirm that vulpinic acid responsive miRNAs act as tumors suppressors for breast cancer.

## Supplementary Information

**Additional file 1: Figure S1.** The quantity of RNA samples for miRNA array assay were showed 1% agarose gel electrophoresis (Line 1-3: MCF-7-Vulpinic acid; Line 4-6: MCF-7-DMSO; Line 7-9: MCF-12A- Vulpinic acid; Line 10-12: MCF-12A-DMSO). **Figure S2.** Normalization of the raw microarray data were performed by using quantile normalizaton method.

## Data Availability

The datasets used and/or analyzed in the current study are available from the corresponding author on reasonable request.

## Abbreviations

3′ UTR: 3′ Untranslated region
ACA: Acetoxychavicol acetate
ATCC: American Type Culture Collection
BAX: BCL2-associated X protein
BCL2: B-cell CLL/lymphoma 2
BRB-Array Tool: Biometric research program
CASP3: Caspase 3, apoptosis-related cysteine protease
CASP9: Caspase 9, apoptosis-related cysteine protease
CI: Cell index
DMEM: Dulbecco’s modified Eagle’s medium
DMSO: Dimethyl sulfoxide
DNA: Deoxyribonucleic acid
EGCG: Epigallacatechingallate
ER: Estrojen receptor
FBS: Fetal Bovine Serum
FOXO 3: Forkhead box O3
GAPDH: Glyceraldehyde-3-phosphate dehydrogenase
GO: Gene Ontology
HER2: Human epidermal growth factor receptor 2
HRP: Horseradish peroxide
JAK/STAT: Janus kinase**-**signal transducer and activator of transcription
JNK: Jun N-terminal kinase
KEGG: Kyoto Encyclopedia of Genes and Genomes
MAPK: Mitogen-activated protein kinase
miRNA: MicroRNA
NCI: Normalized cell index
NF-κB: Nuclear factor kappa B
NM: Nano molar
PARP: Poly (ADP-ribose) polymerase
PBS: Phosphate Buffered Saline
PCR: Polimerase chain reaction
PR: Progesterone receptors
PVDF: Polyvinylidene difluoride
qRT-PCR: Quantitative reverse transcription polymerase chain reaction
RNA: Ribo nucleic acid
RTCA: Real time cell analysis
SDS-PAGE: Sodium dodecyl sulfate–polyacrylamide
VA: Vulpinic acid
