# Supplementary figures and images for: The role of vulpinic acid as a natural compound in the regulation of breast cancer-associated miRNAs
Source: Biol Res. 2021 Nov 7;54:37. doi: 10.1186/s40659-021-00360-4 (PMC8574026; doi:10.1186/s40659-021-00360-4)

**Fig. S1**

**Fig. S2**

**
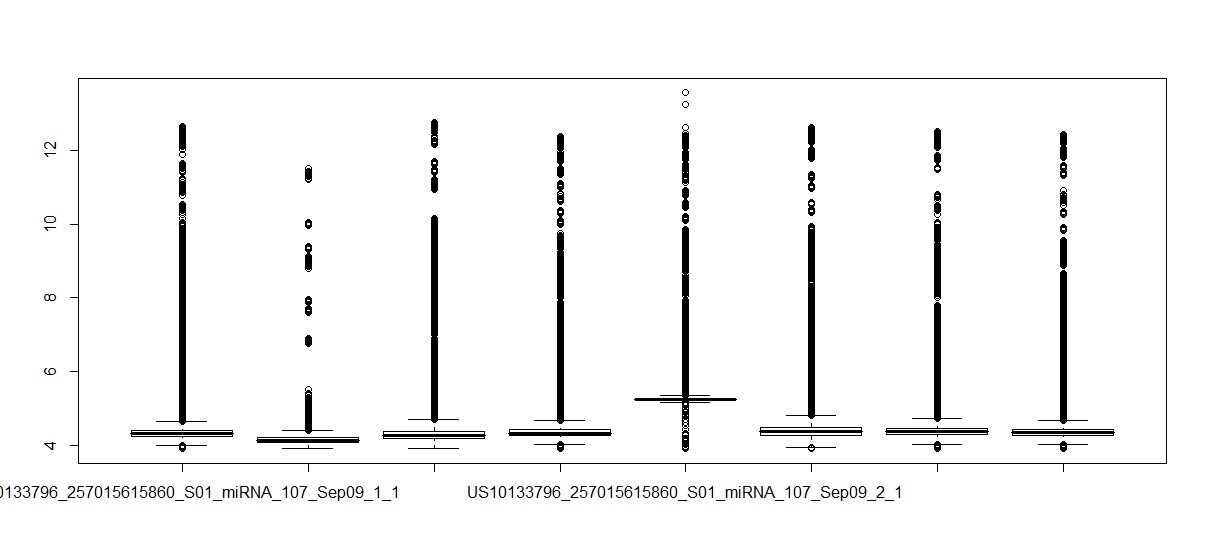
**

**
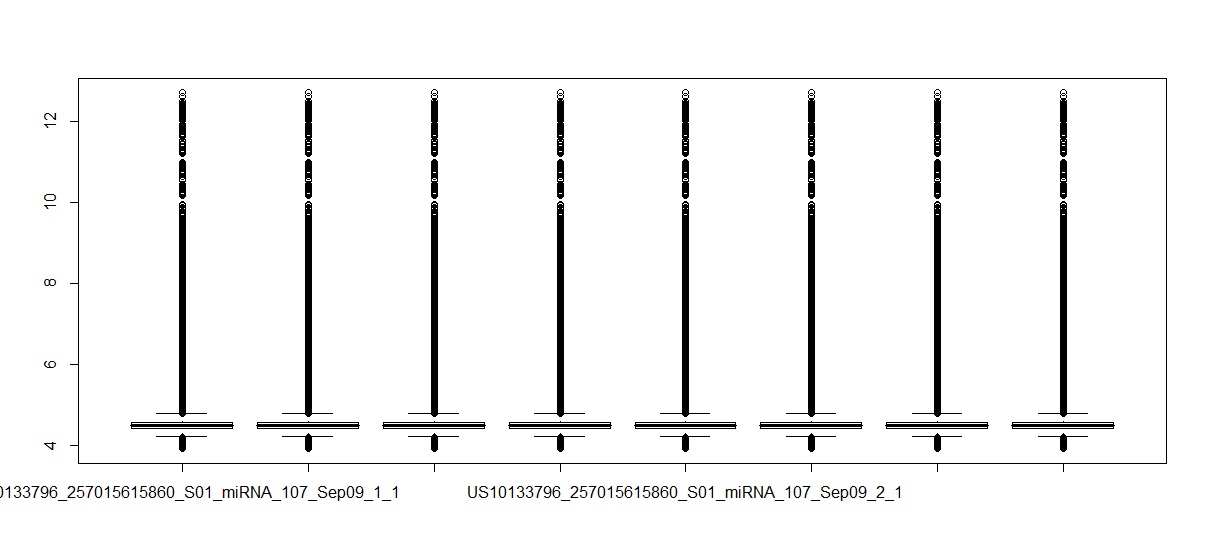
**

Supplement: Supplementary file 1 — Additional file 1: Figure S1. The quantity of RNA samples for miRNA array assay were showed 1% agarose gel electrophoresis (Line 1-3: MCF-7-Vulpinic acid; Line 4-6: MCF-7-DMSO; Line 7-9: MCF-12A- Vulpinic acid; Line 10-12: MCF-12A-DMSO). Figure S2. Normalization of the raw microarray data were performed by using quantile normalizaton method. [file 40659_2021_360_MOESM1_ESM.docx]
